# Differing Prevalence and Diversity of Bacterial Species in Fetal Membranes from Very Preterm and Term Labor

**DOI:** 10.1371/journal.pone.0008205

**Published:** 2009-12-08

**Authors:** Hannah E. Jones, Kathryn A. Harris, Malika Azizia, Lindsay Bank, Bernadette Carpenter, John C. Hartley, Nigel Klein, Donald Peebles

**Affiliations:** 1 Infectious Diseases and Microbiology Unit, Institute of Child Health, London, United Kingdom; 2 Department of Obstetrics and Gynecology, Institute for Womens Health, University College London, London, United Kingdom; 3 Microbiology Department, Camelia Botner Laboratories, Great Ormond Street Hospital, London, United Kingdom; The University of Adelaide, Australia

## Abstract

**Background:**

Intrauterine infection may play a role in preterm delivery due to spontaneous preterm labor (PTL) and preterm prolonged rupture of membranes (PPROM). Because bacteria previously associated with preterm delivery are often difficult to culture, a molecular biology approach was used to identify bacterial DNA in placenta and fetal membranes.

**Methodology/Principal findings:**

We used broad-range 16S rDNA PCR and species-specific, real-time assays to amplify bacterial DNA from fetal membranes and placenta. 74 women were recruited to the following groups: PPROM <32 weeks (n = 26; 11 caesarean); PTL with intact membranes <32 weeks (n = 19; all vaginal birth); indicated preterm delivery <32 weeks (n = 8; all caesarean); term (n = 21; 11 caesarean). 50% (5/10) of term vaginal deliveries were positive for bacterial DNA. However, little spread was observed through tissues and species diversity was restricted. Minimal bacteria were detected in term elective section or indicated preterm deliveries. Bacterial prevalence was significantly increased in samples from PTL with intact membranes [89% (17/19) versus 50% (5/10) in term vaginal delivery p = 0.03] and PPROM (CS) [55% (6/11) versus 0% (0/11) in term elective CS, p = 0.01]. In addition, bacterial spread and diversity was greater in the preterm groups with 68% (13/19) PTL group having 3 or more positive samples and over 60% (12/19) showing two or more bacterial species (versus 20% (2/10) in term vaginal deliveries). Blood monocytes from women with PTL with intact membranes and PPROM who were 16S bacterial positive showed greater level of immune paresis (p = 0.03). A positive PCR result was associated with histological chorioamnionitis in preterm deliveries.

**Conclusion/Significance:**

Bacteria are found in both preterm and term fetal membranes. A greater spread and diversity of bacterial species were found in tissues of women who had very preterm births. It is unclear to what extent the greater bacterial prevalence observed in all vaginal delivery groups reflects bacterial contamination or colonization of membranes during labor. Bacteria positive preterm tissues are associated with histological chorioamnionitis and a pronounced maternal immune paresis.

## Introduction

Preterm delivery before 32 weeks gestation is a leading cause of perinatal mortality and morbidity. The need for an improved understanding of the factors initiating PTL (preterm labour) is underlined by data showing that the number of preterm births is increasing [1]. Of the known risk factors associated with preterm birth, infection is thought to be particularly important. Bacteria from the vagina can access fetal membranes by ascending the cervical canal and then, in some cases, infect amniotic fluid and fetal blood. Alternatively, organisms circulating in maternal blood can cross the placenta and target fetal membranes [2], [3]. Many studies have therefore focussed on the use of antibiotics to treat preterm parturition. In the case of PPROM (preterm prolonged rupture of membranes) administration of antibiotics to women has been shown to delay delivery and reduce neonatal morbidity [4], [5] although antibiotics do not eradicate intra-amniotic infection [6]. In contrast to PPROM the use of antibiotics to prevent preterm labour with intact membranes is of no proven benefit and may be detrimental [5], [7]. It is apparent that a number of findings challenge the assumption that bacteria are the major aetiological factor in preterm delivery.

Bacteria that have been implicated in preterm delivery include commensal bacterial species found in the vaginal tract such as genital mycoplasmas [8]–[11] and *Streptococcus agalactiae* [12], and commensal species of the oral cavity such as *Fusobacterium nucleatum* [3], [13], [14]. However, many earlier studies were based on bacterial identification using culture methodology; but more recent studies suggest that molecular techniques may be more sensitive.

The identification of bacteria by the detection of bacterial DNA has two major advantages. Firstly, the bacteria are not required to grow in culture; this is critical as the fastidious nature of the majority of microorganisms associated with preterm delivery means that culture techniques may under-report the true prevalence. Secondly, even if organisms are no longer viable as a result of antibiotic administration, as is often the case for preterm prolonged rupture of membranes, bacterial DNA may still be detected.

The bacterial 16S ribosomal gene contains regions that are conserved between bacterial species and areas that are variable between species. By using primers that target the conserved regions of the gene it is therefore possible to amplify DNA from most bacterial species. The bacterial DNA is then sequenced to allow identification of the bacteria. The use of broad-range 16S rDNA endpoint PCR has been used most recently to identify bacteria in the amniotic fluid of women with preterm and term labour [13], [14], but there is inconclusive data on the bacterial invasion of fetal membranes and placental tissue. One study failed to identify any bacterial DNA by broad-range 16S rDNA PCR in placental tissue from preterm deliveries [15], whereas another study showed that all fetal membranes, whether from preterm and term deliveries, were positive by broad-range 16S rDNA fluorescent *in situ* hybridisation [3]. The identity and quantity of bacteria in the fetal membranes is likely to be of importance as activation of the innate immune pathway in chorion and decidua is thought to be a key event in the aetiology of preterm birth [16], [17].

The aim of our study was to provide definitive data on the invasion of bacteria in fetal membranes and placental tissue from very preterm and term deliveries using a combination of broad-range 16S rDNA endpoint PCR and a panel of real time PCR assays for the detection on bacterial species previously associated with preterm delivery. We further sought to determine if the presence of bacteria was related to maternal inflammation.

## Methods

### Study population

This study was approved by the University College London/University College Hospital Research Ethics Committee (REC number 03/0179 Brain injury in premature infants: the importance of infection and inflammation) and informed written consent was obtained from all participants. Women were recruited consecutively, with the control groups being recruited over a three month and the preterm groups a longer two year period. Placental tissue and fetal membranes from the following women were studied; those delivering at less than 32 weeks gestation following preterm prolonged rupture of membrane (PPROM) with at least 18 hours between rupture and delivery [(PPROM) n = 26; 11 caesarean section (CS)] or preterm labour with intact membranes [(PTL) n = 19; all vaginal delivery (V)]; those having (prelabour) CS before 32 weeks gestation because of intrauterine growth restriction (indicated preterm birth n = 8) and those having deliveries beyond 37 weeks gestation (term n = 21; 11 CS).

Immediately following delivery the placenta was delivered into a sterile container. Using gamma-irradiated forceps the superficial layer of the amnion was removed to reduce risk of surface contamination. A total of five sections from different parts of the placenta were sampled in a sterile manner with a change of forceps between each sample. These sites were 1 cm^2^ of placental membrane (amnion and chorion) from the centre of the placenta (adjacent to the umbilical cord insertion) and the periphery. In addition, a section of placental parenchyma (without membranes) both from centre and periphery and a segment of umbilical cord were sampled and frozen immediately at −80C for subsequent DNA extraction. Women were considered to have clinical chorioamnionitis if they had a combination of maternal pyrexia of ≥38°C with uterine tenderness and/or offensive vaginal discharge. The placenta was collected routinely in all women delivering before 32 weeks gestation and histological chorioamnionitis was diagnosed if there was polymorph infiltration in both membranes covering the placental disc and peripherally.

### DNA extraction from tissue

Genomic DNA was extracted from 20 mg of placental tissue or fetal membranes using a QIAmp DNA Kit (Qiagen), following the tissue extraction protocol with an additional mechanical lysis step using a Ribolyser cell disrupter (Hybaid). All samples were eluted into a total volume of 200 µl of UV irradiated AE. A negative extraction control (200 ul sterile, UV irradiated) was included in each extraction run.

### Bacterial identification by broad-range 16S rDNA endpoint PCR

PCR was carried out on all DNA extracts using primers based on highly conserved regions of the 16S ribosomal gene. The PCR reaction was as follows, 1 x MolTaq 16S buffer, 1 U MolTaq 16S (all Molzym GmbH & Co), 0.1 mM dNTPs (Invitrogen), 0.4 µM of each of the following primers, Univ 1492 5′ACG GCT ACC TTG TTA CGA CTT 3′ [18], 16SFa 5′ GCT CAG ATT GAA CGC TGG 3′ and 16SFb 5′ GCT CAG GAY GAA CGC TGG 3′ [19] and sterile UV-irradiated water to give a final volume of 45 µl. Extract (5 µl) was added to the PCR reaction and then bacterial DNA was amplified using the following conditions; 94°C×1 min, 36 cycles of 94°C×30 sec, 55°C×30 sec and 72°C×2 min and a final elongation step of 72°C for 5 mins. The sensitivity of this PCR was determined by amplifying DNA from bacterial isolates enumerated as colony forming units. The sensitivity was determined to be ∼10 cfu per reaction for *Escherichia coli* and *Staphlococcus aureus*. Control reactions were carried out for each PCR. A negative control of sterile UV irradiated water (5 µl) was used to assess bacterial contamination of reagents and a PCR inhibition control where 1 µl of *E. coli* DNA (100 cfu/µl) was added along with 5 ul of extracted DNA was also carried out. For all PCR runs two positive controls were used (1 µl of *E. coli* and *S. aureus* both 100 cfu/µl). PCR products (∼1200 bp) were then analysed by agarose gel electrophoresis.

Positive PCR reactions were sequenced with BigDye Terminator v3.1 cycling kit (Applied Biosystems) according to manufacturer's instructions. Briefly, a sequencing reaction was prepared using 1x BigDye sequencing buffer, 1x BigDye ready reaction premix, 0.1 uM primer Univ 1492 or 8A and 3 ul of PCR product for a final volume of 10 ul. The sequencing PCR was cycled under the following conditions: 96°C for 1 min, 25 cycles of 96°C for 10 sec, 50°C of 5 sec, and 60°C for 4 sec. The PCR sequencing reaction was precipitated using ethanol and then analysed on the 3130 Genetic Analyser (Applied Biosystems). All mixed PCR products were cloned using a TOPO-TA PCR cloning kit (Invitrogen). A total of 6 individual clones were amplified directly by PCR using M13 forward and reverse priming sites and six amplicons of the correct size were sequenced as described above using primers T3 or T7. All sequences obtained were then compared to the GenBank database using BLAST program available at National Centre of Biotechnology Information.

### Real-time TaqMan PCR

DNA extracts were further analysed by real time PCR assays for the detection of *Ureaplasma urealyticum, Ureaplasma parvum, Mycoplasma hominis*, *Streptococcus agalactiae* (Group B Streptococci), *Fusobacterium* species and human C reactive protein. Real time species-specific assays have the advantage of being quantitative and are more sensitive than broad range 16S rDNA endpoint PCR. The real-time TaqMan PCR reaction was as follows, 1 x QuantiTect Multiplex PCR master mix (Qiagen), 0.1 µM each of primers and probes, 5 µl of DNA extract and UV irradiated water up to a volume of 40 µl. PCR reactions were analysed using the ABI Prism 7000 detection system (Applied Biosystems). Cycling conditions were as follows: 50°C for 2 min, 95°C for 10 min, and then 45 cycles of 95° for 15 sec and 60°C for 1 min. Negative (U.V irradiated water) and positive controls (100 cfu/ul bacterial DNA) were included in each run. All real-time PCR assays used in this study were validated and optimised to confirm specificity and sensitivity. The sensitivity of each real time assay was established by comparing real time PCR to bacterial culture. Briefly, a bacterial culture was enumerated as colony forming units using the Miles & Misra method [20]. A DNA extract of the bacterial suspension was then prepared by lysing cells with a Ribolyser cell disruptor (Hybaid) followed by centrifugation at 20,000 g for 1 min. The DNA extract was serially diluted and amplified using the real time assay, the sensitivity of each assay was determined to be ∼0.1 cfu per reaction. All samples with a CT threshold below 38 were determined to be positive. All real time primer and probe sequences are shown in Table 1 [21], [22], [23], [24], [25].

**Table 1:** Primers and probe sequences for species-specific real-time PCR assays.

Primer/probe	Sequence	Reference
***Ureaplasma parvum***		
**forward**	**5′CAT TGA TGT TGC ACA AGG AGA AA 3′**	[**21**]
**reverse**	**5′TTA GCA CCA ACA TAA GGA GCT AAA TC 3′**	
**probe**	**FAM 5′ TTG ACC ACC CTT AC GAG 3 TAMRA′**	
***Ureaplasma urealyticum***		
**forward**	**5′ATC GAC GTT GCC CAA GGG GA 3′**	[**21**]
**reverse**	**5′TTA GCA CCA ACA TAA GGA GCT AAA TC 3′**	
**probe**	**JOE 5′TTG TCC GCC TTT ACG AG 3′ TAMRA**	
**Mycoplasma hominis**		
**forward**	**5′ATT GAT TGC TGC AGG TGA TAC A 3′**	[**22**]
**reverse**	**5′GGT GTT ACA ATA TCA GCC CCA AC 3′**	
**probe**	**FAM 5′AGA GCA GCG GCA GTT GAA 3′ TAMRA**	
***Fusobacterium spp.***		
**forward**	**5′GGA TTT ATT GGG CGT AAA GC 3′**	[**23**]
**reverse**	**5′GGC ATT CCT ACA AAT ATC TAC GAA 3′**	
**probe**	**FAM 5′CTC TAC ACT TGT AGT TCC G 3′ TAMRA**	
**Group B Streptococci**		
**forward**	**5′ATC CTG AGA CAA CAC TGA CA 3′**	[**24**]
**reverse**	**5′TTG CTG GTG TTT CTA TTT TCA 3′**	
**probe**	**JOE 5′ATC AGA AGA GTC ATA CTG CCA CTT 3′ TAMRA**	
**Human C reactive protein**		
**forward**	**5′CTT GAC CAG CCT CTC TCA TGC 3′**	[**25**]
**reverse**	**5′TGC AGT AGA CCC CAC CC 3′**	
**probe**	**JOE 5′TTT GGC CAG ACA GGT AAG GGG CAC 3′ TAMRA**	

### Expression of MHC Class II by flow cytometry

Blood was taken from women either in established preterm labour (regular uterine contractions and cervical dilatation of 4 cm or more) or prior to epidural anaesthesia for elective preterm caesarean section. Expression of MHC Class II on blood monocytes was evaluated by staining heparinized blood (10 U/ml) with 5 µg/ml of MHC Class II-FITC and CD14-RPE conjugated monoclonal antibodies for 10 min at room temperature. Blood was then lysed with cell lysis buffer (BD Biosciences) and subsequently washed with FACs buffer (PBS +0.05% sodium azide +0.5% bovine serum albumin). Cells were then fixed with CellFix (BD Biosciences) and then analysed by flow cytometry on a FACScalibur using CellQuest Pro software (BD Biosciences, Oxford, UK). Monocytes form a discrete population when separated by side scatter and forward scatter and are also positive for CD14 expression; this population formed the collection gate and at least 2500 events in this gate were collected for analysis of MHC Class II expression. MHC Class II expression is shown as the median fluorescence intensity (MFI).

### Statistical analysis

The differences in the median value of gestational age, maternal age and birth weight between groups were calculated using the Kruskal-Wallis analysis of variance. For comparison of MHC Class II expression between two groups a Mann Whitney U Test was carried out. Comparisons between proportions were performed by Fisher's exact test. All analysis was carried out on Graphpad Prism version 5.0 or SPSS version 16.0 and a P value of <0.05 was considered significant.

## Results

A total of 74 women were included in this study, the characteristics of each group is shown in Table 2. Twenty-one women (28%) delivered at term and 53 (72%) delivered before 32 weeks of gestation. For those women who delivered before 32 weeks, 19 had PTL with intact membranes (V), 26 delivered preterm with PPROM (11; CS) and 8 had preterm CS deliveries due to intrauterine growth restriction (indicated preterm birth). Maternal age was similar between the groups (p = 0.20). The median gestational age was slightly higher for the indicated preterm birth group (29.7 wk) compared to PTL with intact membranes (25 wk), PPROM (CS) (27.3 wk) and PPROM (V) (25.4 wk) but this was not significant. The median birth weight was lower for the PTL with intact membranes group compared to PPROM (V), PPROM (CS) and the indicated preterm delivery group.

**Table 2:** Summary of data from pregnancies included in this study.

Patient groups	Term (V)	Term (CS)	Indicated preterm delivery (IUGR) (CS)	PTL with intact membranes (V)	PPROM (V)	PPROM (CS)
Number (n)	10	11	8	19	15	11
Delivery method	V	ELCS	ELCS (5) EMCS (3)	V	V	EMCS
Median maternal age (y)	30.5 (22–36)	39(33–47)	33 (21–35)	34 (17–39)	30 (25–46)	35(26–40)
Median gestational age (wk) *P = 0.0001	39.9 (39.1–41.9)	38.9(38–39.9)	29.7 (27–31.3)	25 (22.1–31.1)	25.4(19.7–30.57)	27.3(24.1–32)
Median birth weight (g) *P = 0.0001	3360(2650–3880)	3500(2900–4436)	807.5 (722–1731)	753(451–1600)	840(340–1404)	870(600–2300)
Singleton %	100	100	75	63	80	55
Membrane rupture (d)	0	0	0	0	12 (2–45)	18(2–30)
Chorioamnionitis and/or funisitis n (%)	**	**	0 (0%)	13 (68%)	13 (87%)	9 (82%)

A total of 74 women were recruited into the following groups: term labour V or CS, preterm prolonged rupture of membranes (PPROM) V or CS, preterm labour with intact membranes (PTL) and indicated preterm deliveries (e.g. delivered by CS because of (IUGR) intrauterine growth restriction). The median and interquartile range for maternal age, gestational age, birth weight and membrane rupture are shown.

*Differences between the groups was analysed by the Kruskall Wallis test.

**Histological chorioamnionitis is not routinely assessed in term deliveries. (ELCS) elective caesarean section, (EMCS) emergency caesarean section, (V) vaginal delivery, (CS) caesarean section.

### Prevalence of bacteria in placental tissue and fetal membranes

In order to determine bacterial prevalence during very preterm and term delivery, DNA was extracted from placental tissue and fetal membranes from all women in the study. The DNA extracts were then subject to broad-range 16S rDNA endpoint PCR and real time PCR assays for the detection of *U. parvum, U. urealyticum, M. hominis, Fusobacterium spp.* and *S. agalactiae*. The species-specific real time PCR assays were chosen to detect bacteria that have already been previously associated with preterm birth [2], [3], [8], [10], [12]. To confirm successful DNA extraction a human C reactive protein real time assay was run for each DNA extraction. Human C reactive protein DNA sequence was amplified in all samples and a consistent Ct value was also obtained for each sample.

Following broad-range 16S rDNA PCR a total of 22 (30%) women had fetal membranes and placental tissue positive for bacterial DNA. These samples were further analysed by species-specific real time PCR. 32 (43%) women were positive for bacterial DNA by this method but only 14 of these women were positive using both methods. Women were considered positive if bacterial DNA was isolated, by either method, from at least one sample out of the 5 taken from each women. A complete description of all patients that were positive for bacterial DNA is shown in Figure S1.

**Figure 1:**
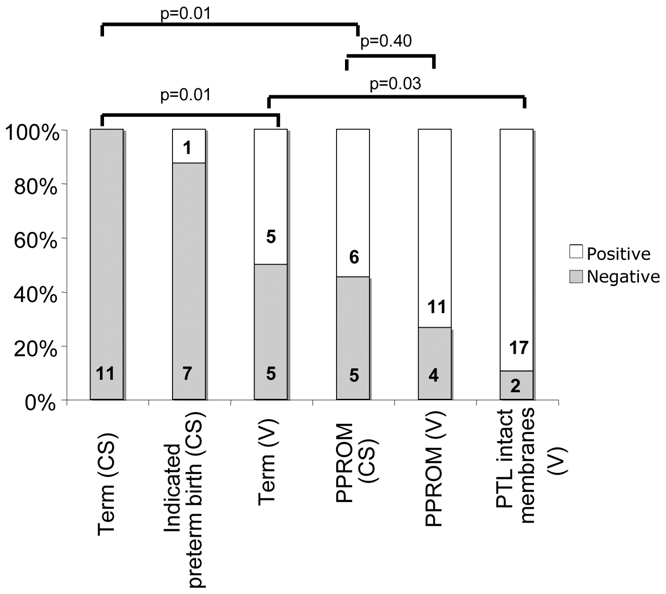
The microbial prevalence in placental tissue and fetal membranes from term and very preterm deliveries. The microbial prevalence was determined in the following groups term with vaginal delivery (V) or caesarean section (CS), indicated preterm delivery, preterm delivery with PPROM, and preterm delivery with intact membranes (PTL). An individual was deemed positive if any of the 5 tissue samples taken were positive for the amplification of bacterial DNA by either method described. The frequency of bacterial prevalence in each group is expressed as a percentage and the number in each group. Groups were compared and significance was determined by a Fisher's exact test.

Figure 1 shows the percentage of women who were positive for bacteria in all groups. No bacteria were detected in samples following CS at term and only 1 individual was positive for bacteria in the indicated preterm group with elective section; however, significantly more women in the term vaginal delivery group were positive for bacteria [(50%) p = 0.01] compared to term CS. Bacterial DNA was detected in over 70% of tissues from women with PPROM who delivered vaginally which did not differ significantly (p = 0.40) from the percentage (55%) in the PPROM group delivering by CS. However, a significant difference was observed between term and PPROM elective section (p = 0.01). There was no correlation between bacterial prevalence and the lengths of time membranes were ruptured in PPROM cases (data not shown). The final group studied were women in spontaneous preterm labour with intact membranes, of which almost 90% were positive for bacteria, which was significantly higher than that seen in term vaginal deliveries (p = 0.03).

**Figure 2:**
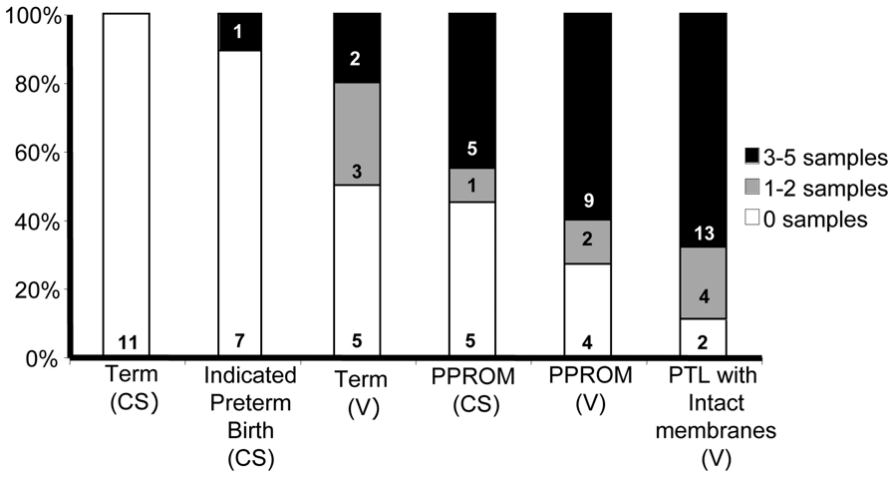
The spread of bacteria in placental tissue and fetal membranes from term and very preterm deliveries. A total of 5 samples were taken from each women. DNA was extracted and then subject to broad-range 16S rDNA endpoint PCR and species-specific real time PCR. The percentage (and number) of individuals that had 3–5 samples, 1–2 samples, and 0 samples positive for bacteria are shown for each group. (CS) caesarean section, (V) spontaneous vaginal delivery, (PTL) preterm labour with intact membranes, (PPROM) preterm prolonged rupture of membranes.

Having established that the frequency of bacterial positive tissue was higher in women with PTL and PPROM, we assessed the extent of bacterial spread by recording the number of samples (maximum of 5, as described in methods) collected from each women that were positive for bacteria. Figure 2 shows that, compared to term groups, more women in the PPROM (45% CS, 60% V) and PTL (68%) groups had more than three positive samples. However, out of the 5 sites sampled there was no one site that was more frequently positive for bacteria (Figure S1). In the term (V) delivery group, where 50% of individuals were found to be positive for bacteria, 80% of the patients had 2 or less samples positive for bacteria. So although bacteria were found in term (V) group there appears to be less bacterial spread throughout the tissue.

### Microbial diversity in fetal membranes and placental tissue

Data were then analysed to determine whether the higher prevalence of bacterial colonisation observed in very preterm samples reflected a greater diversity of bacterial species. Positive PCR products amplified by broad-range 16S rDNA PCR were sequenced and identified by comparing to known bacterial 16S gene sequences. A detailed summary of bacteria identified for each individual case is shown in Figure S1.

Among the 40 patients whose fetal membranes and placental tissue tested positive for bacteria, 25 different bacterial species were identified (Table 3). Most of the bacterial species identified are known to reside in the vaginal, gastrointestinal or respiratory tract. Only a limited number of bacterial species (5), these included healthy vaginal flora (*L. crispatus*) and fecal flora (*E. rectale* and *Pantoea spp*.), were identified in the control group and these were mostly seen in the term (V) group. In contrast there were an array of different bacterial species identified in tissues from PPROM and PTL deliveries. Many of the bacterial species identified, including *U. parvum, Fusobacterium spp, S. agalactiae and S. mitis*, were identified in both PPROM and PTL deliveries. Moreover, all of these bacterial species were found in tissues from more than one woman.

**Table 3:** The bacterial species found in placental tissue and fetal membranes from term and preterm labour.

Group	Bacterial species identified
Term (CS)	
Indicated preterm birth (CS)	*Fusobacterium spp (1)*
Term (V)	*Ureaplasma parvum (2) Lactobacillus crispatus (2), Fusobacterium spp (1) Pantoea spp (1)* and *Eubacterium rectale (1)*
Preterm labour with PROM (CS)	*Ureaplasma parvum (4), Streptococcus mitis group (2), Fusobacterium spp (1), Veillonella parvula (1), Haemophilus influenzae (1) and Ureaplasma urealyticum.*
Preterm labour with PROM (V)	*Ureaplasma parvum (7), Fusobacterium spp (3), Streptococcus agalactiae (2), Mycoplasma hominis (2), Atopobium vaginae (1), Lactobacillus crispatus (1), Eschericha coli (1), Peptoniphilus lacrimalis (1), Corynebacterium amycolatum (1)* and *Ureaplasma urealyticum (1)*
Preterm labour with intact membranes (V)	*Ureaplasma parvum (8), Fusobacterium spp (7*), *Streptococcus agalactiae (4), Streptococcus mitis group (2), Lactobacillus crispatus (2), Haemophilus influenzae (1), Oribacterium sinus (1), Veillonella spp (1), Peptostreptococcus (1), Enterobacter aerogenes (1), Corynebacterium aerogenes (1), Gardnerella vaginalis (1), Finegoldia magna (1), Peptoniphilus asaccharolyticus (1), Streptococcus anginosus (1)* and *Bacteroides ureolyticus*.

The bacterial species found in each group are shown. The incidence of individual species is shown in brackets. (CS) caesarean section, (V) vaginal delivery.

**Figure 3:**
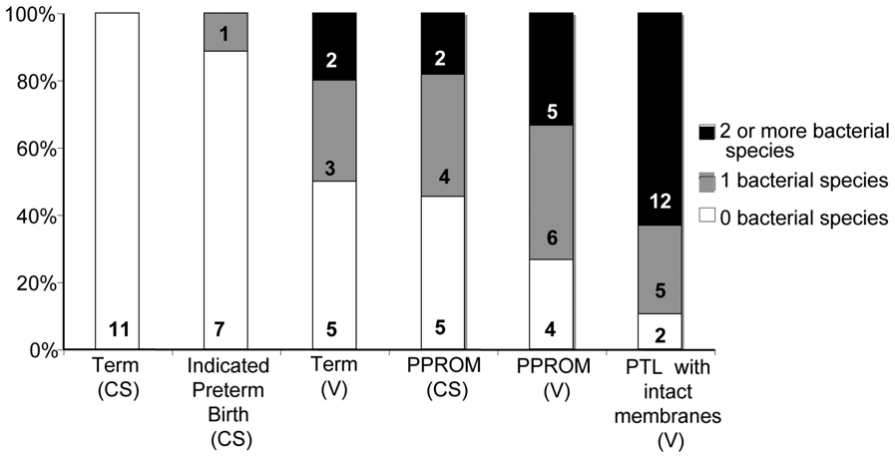
The diversity of bacterial species found in tissues from women with very preterm and term deliveries. The percentage (and number) of women with placental tissue and fetal membranes positive for 2 or more, 1 or 0 bacterial species is shown for each group. (CS) caesarean section, (V) spontaneous vaginal delivery, (PTL) preterm labour with intact membranes, (PPROM) preterm prolonged rupture of membranes.

Mixed infections, where 2 or more bacterial species was identified (Figure 3), were found in over 60% of PTL tissues. Very few mixed infections were identified in term vaginal deliveries (20%), and no mixed infections were observed in term ELCS and indicated preterm delivery.

### The association of bacteria and materno-fetal inflammation

**Figure 4:**
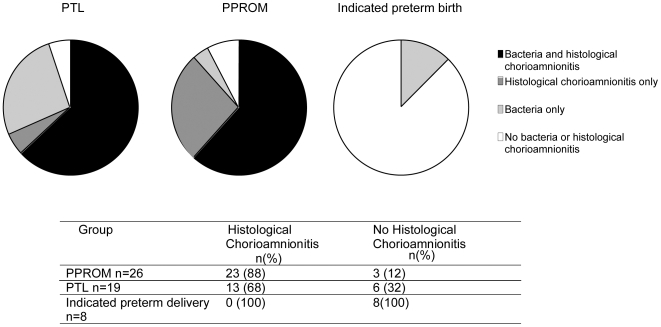
The presence of histological chorioamnionitis in fetal membranes from very preterm deliveries. Fetal membranes of all deliveries before 32 weeks gestation were assessed routinely for histological chorioamnionitis. Here we show the association between the presence of bacteria in fetal membranes and placental tissue and histological chorioamnionitis in preterm labour with intact membranes (PTL), preterm prolonged rupture of membranes (PPROM), and indicated preterm delivery.

It is widely thought that inflammation plays a critical role in the initiation of labour. To determine the inflammatory status at the site where we identified the bacteria we assessed the presence of chorioamnionitis in very preterm fetal membranes (Figure 4). In indicated preterm delivery there was a complete absence of histological chorioamnionitis, in contrast PPROM and PTL groups had significantly higher incidence of histological chorioamnionitis (88% and 68% respectively). Membranes that were positive for bacteria were more likely to also demonstrate histological chorioamnionitis than those without [Odds Ratio (OR) 5; 95% CI, 1.43 to 17.38]. However there were a number of cases in the PPROM and PTL groups that were positive for either bacteria or histological chorioamnionitis but not for both.

**Figure 5:**
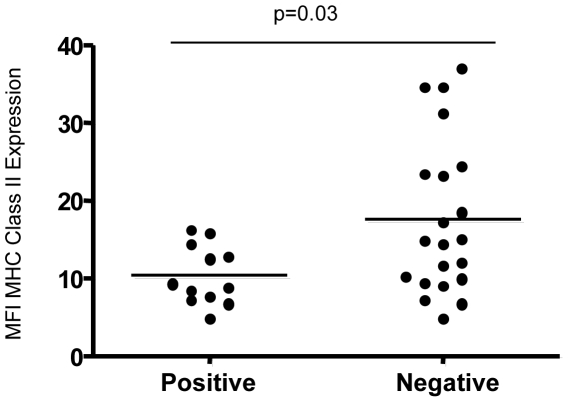
Monocyte MHC Class II expression in women with very preterm labour and preterm premature rupture of membranes with and without the presence of bacteria. Maternal monocytes were analysed for the expression (median fluorescence intensity- MFI) of MHC Class II by flow cytometry. The differential expression of MHC Class II expression in very preterm deliveries (PTL with intact membranes and PPROM CS and V; n = 33 as only 33 blood samples were processed out of the possible 45 total of PPROM and PTL groups) with or without the presence of bacteria (as defined by positive bacterial DNA amplification by broad-range 16S rDNA PCR only) was analysed by Mann-Whitney U test.

We have previously shown that term labour is associated with a fall in MHC Class II expression on maternal blood monocytes, but this reduction is more pronounced in PTL and PPROM groups [26]. This fall in MHC Class II expression is known as monocyte hyporesponsiveness and may provide insight into immune paresis [27], [28]. We therefore went on to determine whether there was an association between maternal monocyte MHC class II expression and bacterial presence in placental tissue and membranes (Figure 5 and Figure S2). There was a significant reduction of MHC Class II expression, as indicated by median fluorescence intensity (MFI), in those women who were positive for the bacterial ribosomal 16S gene by broad-range 16S rDNA endpoint PCR (p = 0.03).

## Discussion

These data demonstrate a number of important points: 1) addition of real-time species specific PCR to broad-range 16S rDNA endpoint PCR increases the detection rate of bacteria in placental samples 2) fetal placenta and membranes are more likely to contain bacterial DNA following PTL and PPROM, and the diversity of bacterial species is also increased in these groups 3) bacterial presence in the membranes correlates with evidence of an inflammatory response in both maternal blood and the membranes themselves 4) compared to recent studies using similar PCR based techniques [13], [14] bacterial infection of the membranes is more common than the amniotic fluid of women who deliver preterm.

The development of molecular techniques for detection of bacterial DNA in tissues has made it clear that bacterial culture methods underestimate the prevalence of bacteria in placenta and amniotic fluid [29]. Yoon and colleagues [30] showed a *U. urealyticum* PCR increased the detection of *U. urealyticum* by almost 40% compared to using standard culture methods. Similarly Han *et al* [14] showed that molecular techniques not only increased the detection rate of intraamniotic infection but also the diversity of bacterial species identified and the presence of mixed infection. However not all studies have reported high rates of bacterial colonisation using molecular techniques; the failure to amplify bacterial DNA in placental tissue [15] in one such study highlights the effect of different methodologies. We therefore optimised the protocol in our study to improve bacterial detection. In addition to sampling a total of 5 sites from each placenta, a combination of broad-range 16S rDNA endpoint PCR and species-specific real time PCR was used. The species-specific real time assays used in this study are a log more sensitive than broad range 16S methodology; this allowed us to detect certain species at much lower numbers than could be achieved by broad range methods alone. In future it may therefore be useful to run panels of species-specific real time assays, although additions to our panel would be needed as not all 16S rDNA positive tissue was also positive by species-specific real time. This approach has been successful in bacterial vaginosis and periodontal disease [23], [31]. To further increase the probability of identification of mixed infections all positive DNA products amplified by broad-range 16S rDNA PCR were cloned and each clone was sequenced (as described in the methods). Finally, we included relevant controls; patient cohorts included term and preterm deliveries that were delivered by caesarean section and vaginal delivery and all PCR assays included controls for PCR inhibition and insufficient DNA extraction.

Using this approach we identified bacterial DNA from 25 different species, with 90% of PTL with intact membranes, 73% of PPROM (V) and 55% PPROM (CS) cases having at least one positive sample. All organisms reported have been previously identified in the tissues or amniotic fluid from women with preterm labour although the prevalence is particularly high in this study. As with other studies the most common species identified, in this study by species-specific real time PCR, were *U. parvum* closely followed by *Fusobacterium spp* [3], [13], [32]. A large proportion of bacteria identified typically reside in the vaginal tract, including *U. parvum, S. agalactiae, M. hominis* and *A. vaginae*. These bacterial species are also associated with bacterial vaginosis, a change in the vaginal flora, which has been linked to preterm birth [11], [32], [33]. Other microorganisms include *Fusobacterium spp., S. mitis* and *H. influenzae*, all of which are most commonly found in the upper respiratory tract. It is hypothesised that these organisms gain access to the placenta via haematogenous spread from the oral cavity [34].

The observation that bacteria are more prevalent in women delivered vaginally, both very preterm and at term, compared with delivery by caesarean section suggests that the process of labour and vaginal delivery increase the prevalence of bacteria; there are several ways that this could happen. Firstly, although the samples were collected meticulously to avoid contamination by vaginal flora, this might have occurred. It is however unlikely that contamination contributes significantly to the findings for the following reasons:1) the greater bacterial spread and species diversity observed in all preterm groups, compared with term vaginal delivery, is consistent with an aetiological role for bacteria in preterm birth 2) very few *Lactobacillus* species were identified in the vaginal deliveries (Term (V) 2/10; PTL (V) 2/19; PPROM (V) 0/10). As *Lactobacillus* spp. is the predominant species observed in the vagina, if contamination had occurred, then it would most likely have been with this organism 3) it was not possible to show that any particular sample site was more likely to be positive; if contamination were the major cause for bacterial presence then it would be predicted that peripheral membrane would be more likely to be more positive than placental parenchyma. Secondly, recent data showing a higher frequency of intra-amniotic infection in women in active labour, compared to caesarean section, suggest that these bacteria may have been present from early in the labour process and that labour itself can result in colonisation of the membranes [35]. In addition, other studies have shown bacterial colonisation of term pregnancies, including those delivered by caesarean section suggesting that bacterial colonisation does not necessarily result in preterm birth [36].

We found a similar frequency and diversity of bacteria in both the PPROM and PTL with intact membrane groups. Conventional teaching would be that chorioamnionitis and the presence of intrauterine bacteria should be more common following PPROM both because bacteria are thought to cause rupture of membranes and because removal of the membrane barrier makes the uterine contents more accessible. In addition, a recent study reported a lower rate of bacterial infection in PTL groups than PPROM [3], although the broad-range 16S PCR used may not have been particularly sensitive as only 6 different bacterial species were identified (cf. 25 in our study). In contrast in our study, the trend was to lower rates of bacterial colonisation following PPROM than PTL, although the prevalence of chorioamnionitis was higher in PPROM groups compared to the PTL group. There are a number of possible explanations for these results; 1) The presence of bacteria does not always result in an inflammatory response, this may be due to brief bacterial colonisation of the membranes that has not resulted in an established infection; 2) The use of a combination of a broad-range 16S rDNA PCR and species-specific real time PCR in this study has increased the detection rate in the PTL group. It is possible that the universal treatment of women with PPROM, but not PTL, with erythromycin reduced the bacterial DNA load below the detection limits in some cases and so underestimated the role of bacteria in this group. Despite this it is important to note that following antibiotic therapy in the PPROM (V) and PPROM (CS) groups 73% and 55% of cases respectively were bacteria positive. Although a study by Gomez and colleagues showed that antibiotic administration to women with PPROM resulted no change in intra-amniotic infection [6], but to our knowledge no studies have specifically looked at fetal membranes.

These data strongly support the concept that bacteria play an important role in many cases of very preterm delivery. The diversity and number of positive samples per case was higher in the preterm (PPROM V, PPROM CS, PTL V) groups than term groups (term CS, term V). Fascinatingly, over 60% of PTL cases having 2 or more bacterial species. It is not possible to say however whether it is particular bacterial species or overall bacterial load that is most important. It is perhaps not conceptually surprising that the majority of molecular studies report lower bacterial prevalence in amniotic fluid from PTL than we have shown in the fetal membranes [13], [14]. Particularly for bacteria accessing the uterine cavity from the vagina, the membranes represent the first point of contact and it is at the chorio-decidual interface that interaction with the innate immune system occurs. Pattern recognition molecules such as Toll-like receptors are thought to be important mediators in the host response to bacterial products [37] and it is relevant that chorion and decidua contain the full range of TLR subtypes allowing activation by different bacterial species and even DNA [38], [39]. This is the likely explanation for the correlation observed in our study between bacterial presence and chorioamnionitis. However, although the data in Figure 4 shows that a similar proportion of the PTL with intact membranes and PPROM groups had both bacteria and chorioamnionitis 25% of cases in the PPROM group had chorioamnionitis but no bacteria, whilst approx 25% of the PTL group had bacteria but no chorioamnionitis. We cannot be sure of the reasons for this discordancy but the PPROM group might be influenced by the antibiotics given prior to birth, reducing the bacterial load below the PCR sensitivity whilst in the PTL group the bacteria were non pathological, contaminants (see below) or hadn't yet had time to trigger a typical chorioamnionitis.

We and others have shown evidence of a maternal systemic inflammatory response in association with preterm labour with increased pro-inflammatory cytokines and decreased monocyte MHC Class II expression in maternal blood [26], [40]–[43]. The observation that monocyte MHC Class II expression is also lower in women whose placenta was bacteria positive suggests that bacterial presence in membranes may contribute to the maternal, as well as chorio-decidual, inflammatory response. However, there is an overlap between the two groups with some women with no evidence of bacterial invasion having low Class II expression; bacteria may well not be the only inflammatory trigger and other stimuli, such as CRH have been proposed [44], [45].

There are several factors that could interfere with our stated aim of providing definitive data on the invasion of bacteria in fetal membranes and placenta. First, as discussed previously, bacteria detected in samples from vaginal deliveries may represent contamination of the tissue during its passage through the vagina, rather than bacterial colonisation predating the time of delivery. A second factor that could lead to underestimation of bacterial presence in placental samples is that not all 16S positive tissue was also positive by species-specific real time. This means that although a future approach might be to improve sensitivity by running panels of species-specific real time assays, additions to the panel would be needed; a similar approach has been successful in bacterial vaginosis and periodontal disease [23], [31].

In conclusion, we detected the presence of bacteria in fetal membranes and placenta in 90% of PTL and 65% of PPROM deliveries. However the mere presence of bacteria may be insufficient to precipitate preterm labour. It appears that bacterial diversity, bacterial load and the extent of tissue infiltration in combination with the maternal response to the bacteria may be important. It remains unclear if bacteria are responsible for the maternal inflammatory response or the latter facilitates bacterial infection. Further work is required to dissect the precise roles of infection and inflammation in preterm labour.

## Supporting Information

Figure S1 A summary of bacterial species detected in placental tissue and fetal membranes. Those species shown in bold were detected by species-specific real-time PCR and the rest were detected by broad-range 16S rDNA endpoint PCR. Those bacterial species indicated with an asterix (*) were taken out of analysis as these are likely to be contaminates introduced by reagents or during sampling. The number of samples positive out of 5 (see methods) are shown in brackets. (CS) caesearan section, (V) vaginal delivery. (A) Placental membranes - centre of placenta. (B) Placental parenchyma - centre of placenta. (C) Placental membranes - periphery. (D) Placental parenchyma - periphery. (E) Umbilical cord.
(0.11 MB DOC)

Figure S2 Monocyte MHC Class II expression in women with very preterm labour and preterm premature rupture of membranes with and without the presence of bacteria. Maternal monocytes were analysed for the expression (median fluorescence intensity- MFI) of MHC Class II by flow cytometry. The differential expression of MHC Class II expression in very preterm deliveries (PTL with intact membranes and PPROM CS and V n = 33; PTL and PPROM V only n = 21) with or without the presence of bacteria (as defined by positive bacterial DNA amplification by broad-range 16S rDNA PCR only) was analysed by Mann-Whitney U test. (CS) caesarean section, (V) vaginal delivery.
(0.53 MB TIF)
