# Supplementary material for: Differing Prevalence and Diversity of Bacterial Species in Fetal Membranes from Very Preterm and Term Labor
Source: PLoS One. 2009 Dec 8;4(12):e8205. doi: 10.1371/journal.pone.0008205 (PMC2785424; doi:10.1371/journal.pone.0008205)
Supplement: Figure S1 — A summary of bacterial species detected in placental tissue and fetal membranes. Those species shown in bold were detected by species-specific real-time PCR and the rest were detected by broad-range 16S rDNA endpoint PCR. Those bacterial species indicated with an asterix (*) were taken out of analysis as these are likely to be contaminates introduced by reagents or during sampling. The number of samples positive out of 5 (see methods) are shown in brackets. (CS) caesearan section, (V) vaginal delivery. (A) Placental membranes - centre of placenta. (B) Placental parenchyma - centre of placenta. (C) Placental membranes - periphery. (D) Placental parenchyma - periphery. (E) Umbilical cord. (0.11 MB DOC) [file pone.0008205.s001.doc]

| **Case Number** | **Number of samples positive for bacteria *** | **Bacteria identified** |
| --- | --- | --- |
| **Term (V)** | | |
| 1 | 3 BCD | *Lactobacillus crispatus* (3) |
| 2 | 1 C | *Lactobacillus crispatus* (1) ***Ureaplasma parvum* (1)** |
| 3 | 1 E | *Pantoea spp* (1)*, Eubacterium rectale* (1) |
| 4 | 1 B | ***Ureaplasma parvum* (1)** |
| 5 | 1 ABCD | ***Fusobacterium spp* (4)** |
| **Term (CS)** | | |
| 1 | 1 | **Oscillatoria spp* (1) |
| 2 | 2 | **Acintobacter iwoffii* (2) |
| **Indicated preterm delivery – delivered due to Intrauterine Growth Restriction (CS)** | | |
| 1 | 1 | **Propionibacterium acnes* (1)*, *Staphlococcus spp* (1) **Acinetobacter spp* (1) |
| 2 | 4 ABCD | ***Fusobacterium spp* (4)** |
| **Preterm labour with intact membranes (V)** | | |
| 1 | 3 CDE | *Streptococcus agalactiae (1)* ***Streptococcus agalactiae (3)*** |
| 2 | 3 BCD | *Lactobacillus crispatus (2),* ***Fusobacterium spp (2)*** |
| 3 | 2 CE | *Streptococcus mitis group (1), Haemophilus influenzae (1), Oribacterium sinus (1)* |
| 4 | 4 BCDE | **Staphlococcus warneri (1), *Propionibacterium acnes (1),* ***Ureaplasma parvum (4).*** |
| 5 | 1 A | *Veillonella spp (1), Peptostreptococcus spp (1),* |
| 6 | 5 ABCDE | **Bacillus subtilis (1), *Paracoccus yeei (1),*  ***Fusobacterium spp (5).*** |
| 7 | 5 ABCDE | *Streptococcus agalactiae* (1), *Enterobacter aerogenes (4),* ***Ureaplasma parvum (5), Streptococcus agalactiae* (1)** |
| 8 | 5 ABCDE | *Lactobacillus crispatus (1), *Pseudomonas spp (2),* ***Ureaplasma parvum (5)*** |
| 9 | 1 E | *Corynebacterium ammoniagenes (1), *Paracoccus spp (1) *Massilia aerolata (1)* |
| 10 | 3 BDE | *Gardnerella vaginalis (2) Finegoldia magna (1), *Staphlococcus spp (1)* |
| 11 | 5 ABCDE | *Streptococcus mitis group (3), Peptoniphilus asaccharolyticus (1),* ***Fusobacterium spp (5)*** |
| 12 | 1 A | ***Fusobacterium spp (1)***, ***Ureaplasma parvum (1)*** |
| 13 | 4 ABCD | ***Ureaplasma parvum* (4)** |
| 14 | 3 ABC | ***Ureaplasma parvum (3), Fusobacterium spp (3)*** |
| 15 | 5 ABCDE | *Streptococcus anginosus (2), Bacteroides ureolyticus (1),* ***Fusobacterium spp (5).*** |
| 16 | 3 ABE | ***Ureaplasma parvum (3), Streptococcus agalactiae* (3)** |
| 17 | 3 ABE | ***Stretococcus agalactiae (1), Ureaplasma parvum (2) Fusobacterium spp (1)*** |
| **Preterm Delivery with PROM (V)** | | |
| 1 | 5 ABCDE | *Ureaplasma parvum (1),* ***Ureaplasma parvum (5)*** |
| 2 | 5 ABCDE | *Streptococcus agalactiae* (1) , *Atopobium vaginae (1),* ***Ureaplasma parvum (5), Fusobacterium spp (5), Streptococcus agalactiae (5).*** |
| 3 | 5 ABCDE | **Pseudomonas spp (1),* ***Ureaplasma parvum (4)*** *Lactobacillus crispatus (1)* |
| 4 | 1 A | **Stenotrophomas maltophilia(1),* ***Fusobacterium spp (1)*** |
| 5 | 2 CD | *Escherichia coli (2)* |
| 6 | 4 ABCD | **Propionibacterium acnes(1)* ***Ureaplasma parvum (4)*** |
| 7 | 5 ABCDE | ***Ureaplasma parvum (5), Mycoplasma hominis (1)*** |
| 8 | 4 ABCD | *Peptoniphilus lacrimalis (1), *Stenotrophomas maltophilia (1), Corynebacterium amycolatum (1),* ***Ureaplasma parvum (4)*** |
| 9 | 5 ABCDE | *Ureaplasma parvum (3)* ***, Ureaplasma parvum (5)*** |
| 10 | 5 ABCDE | ***Ureaplasma urealyticum(2), Fusobacterium spp (5), Streptococcus agalactiae (5)*** |
| 11 | 5 ABCDE | ***Mycoplasma hominis (5)*** |
| **Preterm delivery wth PROM (CS)** | | |
| 1 | 5 ABCDE | **Acinetobacter iwoffii (1),* ***Ureaplasma parvum (5)*** |
| 2 | 1 D | *Streptococcus mitis group (1), Veillonella parvula (1)* |
| 3 | 5 ABCDE | *Ureaplasma parvum (3),* ***Ureaplasma parvum (5)*** |
| 4 | 5 ABCDE | *Haemophilus influenzae (4), Streptococcus mitis group (1)* ***Ureaplasma parvum (5), Fusobacterium spp (5)*** |
| 5 | 5 ABCDE | ***Ureaplasma urealyticum (5)*** |
| 6 | 5 ABCDE | ***Ureaplasma parvum (5)*** |
